# Inflammation following acute myocardial infarction: Multiple players, dynamic roles, and novel therapeutic opportunities

**DOI:** 10.1016/j.pharmthera.2018.01.001

**Published:** 2018-06

**Authors:** Sang-Bing Ong, Sauri Hernández-Reséndiz, Gustavo E. Crespo-Avilan, Regina T. Mukhametshina, Xiu-Yi Kwek, Hector A. Cabrera-Fuentes, Derek J. Hausenloy

**Affiliations:** a Cardiovascular and Metabolic Disorders Program, Duke-National University of Singapore, Singapore; b National Heart Research Institute Singapore, National Heart Centre Singapore, Singapore; c The Hatter Cardiovascular Institute, Institute of Cardiovascular Science, University College London, UK; d The National Institute of Health Research University College London Hospitals Biomedical Research Centre, UK; e Institute of Biochemistry, Medical School, Justus-Liebig University, Giessen, Germany; f Escuela de Ingenieria y Ciencias, Centro de Biotecnologia-FEMSA, Tecnologico de Monterrey, Monterrey, NL, Mexico; g Institute of Fundamental Medicine and Biology, Kazan (Volga Region) Federal University, Kazan, Russian Federation; h German Center for Lung Research, Excellence Cluster Cardio-Pulmonary System, Universities of Giessen and Marburg Lung Center, Giessen, Hessen, Germany; i Yong Loo Lin School of Medicine, National University Singapore, Singapore; j Barts Heart Centre, St Bartholomew's Hospital, London, UK

**Keywords:** ACS, Acute coronary syndrome, AMI, Acute myocardial infarction, AGEs, Advanced glycation end-products, BAFF, B-cell activating factor, C1-INH, C1-inhibitor, CCL2, Chemokine ligand 2, CCL5, Chemokine ligand 5, CCR2, Chemokine receptor 2, CCR5, Chemokine receptor 5, CCR9, Chemokine receptor 5, CR1, Complement receptor 1, CINC-1, CXCL1, GRO α, KC, Cytokine-induced neutrophil chemoattractant 1, DAMPs, Damage-associated molecular patterns, ECM, Extracellular matrix, EDA, Extra domain A, eRNA, Extracellular ribonucleic acids, FN-EDA, Fibronectin-end domain A, HSPs, Heat shock proteins, hs-CRP, High-sensitivity C-reactive protein, HMGB1, High mobility group box 1, ICAM-1/ICAM-2, Intercellular adhesion molecule, IFN-γ, Interferon-γ, IRF5, Interferon regulatory factor 5, IHD, Ischemic heart disease, IL-1, Interleukin-1, IL-8, CXCL8, Interleukin-8, LV, Left ventricular, LTB4, Leukotriene B4, MIP-2α, CXCL2, GRO β, Macrophage inflammatory protein-2α, MMPs, Matrix metalloproteinases, MCP-1, Monocyte chemoattractant protein-1, MyD, Myeloid differentiation primary response gene, MI, Myocardial infarction, NO, Nitric oxide, NLRs, NOD-like receptors, NF-κB, Nuclear factor kappa-light-chain-enhancer of activated B cells, NLRP3, Nucleotide-binding oligomerization domain-like receptor family of cytosolic proteins, PRRs, Pattern recognition receptors, PS, Phosphatidylserine, PMN, Polymorphonuclear leukocytes, PPCI, Primary percutaneous coronary intervention, ROS, Reactive oxygen species, RAGE, Receptor for advanced glycation end-products, Tregs, Regulatory T cells, STEMI, ST segment elevation myocardial infarction, TLRs, Toll-like receptors, TGF-β, Transforming growth factor-β, TNFα, Tumor necrosis factor-alpha, Inflammation, Acute myocardial ischemia and reperfusion injury, Acute myocardial infarction, Monocytes, Macrophages, Lymphocytes, Dendritic cells, Cytokines, Chemokines, Innate immunity

## Abstract

Acute myocardial infarction (AMI) and the heart failure that often follows, are major causes of death and disability worldwide. As such, new therapies are required to limit myocardial infarct (MI) size, prevent adverse left ventricular (LV) remodeling, and reduce the onset of heart failure following AMI. The inflammatory response to AMI, plays a critical role in determining MI size, and a persistent pro-inflammatory reaction can contribute to adverse post-MI LV remodeling, making inflammation an important therapeutic target for improving outcomes following AMI. In this article, we provide an overview of the multiple players (and their dynamic roles) involved in the complex inflammatory response to AMI and subsequent LV remodeling, and highlight future opportunities for targeting inflammation as a therapeutic strategy for limiting MI size, preventing adverse LV remodeling, and reducing heart failure in AMI patients.

## 1 Introduction

Acute myocardial infarction (AMI) and the heart failure that often follows, are among the leading causes of death and disability worldwide. Following an AMI, the most effective treatment for minimizing acute myocardial ischemia/reperfusion injury (IRI), salvaging viable myocardium, and limiting myocardial infarct (MI) size, is timely myocardial reperfusion using primary percutaneous coronary intervention (PPCI). However, the process of myocardial reperfusion, can paradoxically, in itself, induce cardiomyocyte death and myocardial injury, a phenomenon which has been termed ‘myocardial reperfusion injury’, and which can contribute up to 50% of the final MI size (Yellon & Hausenloy, 2007). As such, the mortality and morbidity following AMI remain significant with 7% mortality and 22% death at one year, respectively (Cung et al., 2015). Novel therapies are therefore required to reduce MI size, and prevent adverse LV remodeling in order to reduce the onset of heart failure and improve clinical outcomes following AMI (Cabrera-Fuentes, Alba-Alba, et al., 2016; Cabrera-Fuentes, Aragones, et al., 2016; Hausenloy, Botker, et al., 2017; Hausenloy, Garcia-Dorado, et al., 2017; Preissner, Boisvert, & Hausenloy, 2015).

The inflammatory response to acute IRI plays a critical role in determining acute MI size and subsequent post-MI adverse LV remodeling, making it a potential therapeutic target for improving clinical outcomes in AMI patients. However, the precise role inflammation plays in the setting of AMI has been debated since the 1980s with the infiltration of leukocytes or granulocytes being recognized as inflammatory triggers, as opposed to being ‘innocent’ bystanders of the inflammatory reaction (Engler & Covell, 1987; Engler, Dahlgren, Morris, Peterson, & Schmid-Schonbein, 1986; Engler, Dahlgren, Peterson, Dobbs, & Schmid-Schonbein, 1986). In the 1990s, experimental studies questioned the role of leucocytes as mediators of cardiomyocyte death, suggesting they may play only a minor role in reperfusion injury (Mullane & Engler, 1991; Ritter, Wilson, Williams, Copeland, & McDonagh, 1995; Suematsu et al., 1994). However, there is an increasing amount of experimental evidence, that a number of different players are involved in the inflammatory response, and they have been shown to contribute to the detrimental effects of acute myocardial IRI, making them important therapeutic targets for cardioprotection. In this article we provide an overview of the multiple players (and their dynamic roles involved) in the complex inflammatory response to AMI and subsequent LV remodeling, and highlight future opportunities for targeting inflammation as a therapeutic strategy for limiting MI size and preventing heart failure following AMI.

## 2 The inflammatory response to acute myocardial IRI

**Fig. 1:**
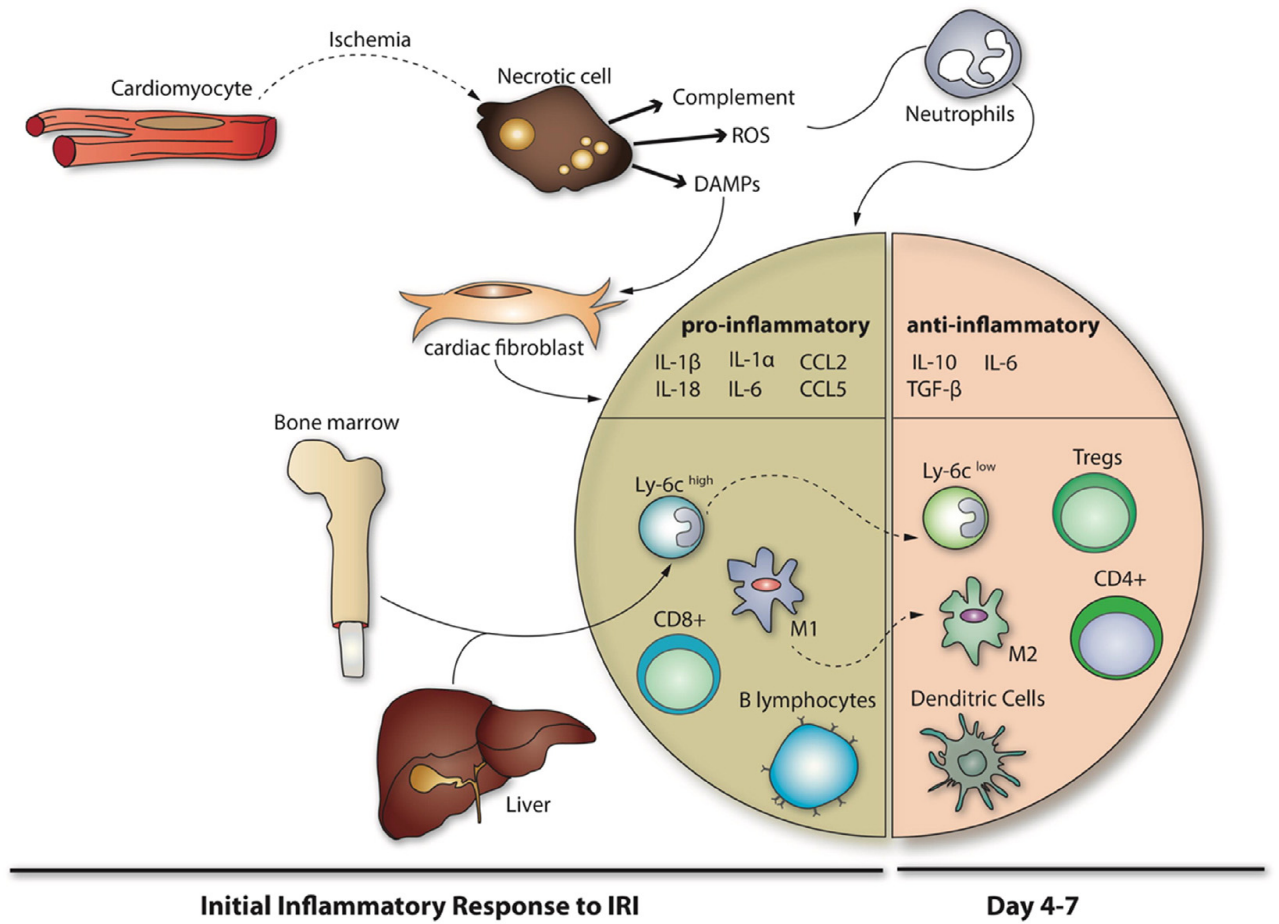
Overview of the inflammatory response to acute myocardial infarction. This schema depicts the initial pro-inflammatory and the subsequent anti-inflammatory reparative phase following AMI. Dying cardiomyocytes during acute myocardial ischemia induce the pro-inflammatory response through the production of DAMPS, ROS, and complement, which through the release of cytokines (such as IL-1β, IL-18, IL-1α, IL-6, CCL2, CCL5), mediate the accumulation of a variety of cells including neutrophils, monocytes, macrophages, B lymphocytes and CD8^+^ T cells into the infarct zone. The subsequent anti-inflammatory reparative phase, mediates the resolution of the inflammatory response through the production of anti-inflammatory factors (such as IL-10, IL6, TGF-β), and changes in monocytes and macrophages, and the recruitment of Tregs, CD4^+^ T cells and dendritic cells.

The onset of acute myocardial ischemia in the setting of an AMI, induces an initial pro-inflammatory response, the purpose of which is to remove necrotic cell debris from the MI zone. The onset of myocardial reperfusion following PPCI, then exacerbates this pro-inflammatory response and contributes to the cardiomyocyte death and myocardial injury characteristic of ‘myocardial reperfusion injury’ which manifests between 6 and 24 h post-reperfusion (Zhao et al., 2000, Zhao et al., 2001). The initial pro-inflammatory response is then followed by an anti-inflammatory reparative phase which allows wound healing and scar formation to occur thereby preventing cardiac rupture. The transition between these two phases is orchestrated by a finely regulated, but complex interaction, between multiple players within the heart itself (including cardiomyocytes, endothelial cells, fibroblasts, and the interstitium), and components of the immune response (including neutrophils, monocytes, macrophages, dendritic cells and lymphocytes) (Fig. 1). Perturbations in both the balance and transition between the pro-inflammatory and the anti-inflammatory reparative phases can exacerbate acute myocardial IRI and contribute to post-MI adverse LV remodeling.

### 2.1 The initial pro-inflammatory response to AMI

Following AMI, the onset of acute myocardial ischemia induces cellular injury and death to different constituents of the myocardium (cardiomyocytes, endothelial cells, fibroblasts and interstitium). This in turn, initiates an acute pro-inflammatory response through the concerted action of several processes including complement cascade activation, reactive oxygen species (ROS) production, and damage-associated molecular patterns (DAMPs) which serve as ligands for pattern recognition receptors (PRRs), such as Toll-like receptors (TLRs) and nucleotide-binding oligomerization domain-like receptor family of cytosolic proteins (NLRP3, also known as Nod-like receptors) inflammasomes. These result in the release of a variety of pro-inflammatory mediators (such as cytokines and chemokines), which induce the recruitment of inflammatory cells into the MI zone, and augment the pro-inflammatory response following AMI. By targeting the viable border zone of the infarction, infiltrating leukocytes may induce the death of cardiomyocytes, thereby extending ischemic injury beyond the original MI zone.

#### 2.1.1 Complement cascade

Activation of the complement cascade contributes to the acute pro-inflammatory response following AMI (reviewed in Timmers et al., 2012). It comprises 30 proteins and protein fragments, many of which are circulating as pro-enzymes and are activated by proteases, through 3 different pathways — classical, lectin and alternate, in response to DAMPs and the release of cardiomyocyte contents during AMI (Timmers et al., 2012). These 3 pathways converge on the common (terminal) complement pathway and result in: (1) opsonization (the process by which a pathogen is marked for ingestion and eliminated by a phagocyte) and phagocytosis to clear foreign and damaged material (complement C3b); (2) inflammation to attract additional phagocytes (complement C3a, C4a, C5a), (3) activation of the cell-killing membrane attack complex (complement C5b-9). The activation of the complement cascade is regulated by various proteins, including C1-inhibitor (C1-INH) and complement receptor 1 (CR1).

A number of experimental small and large animal AMI studies have shown that MI size can be reduced by genetic or pharmacological inhibition of various components of the complement cascade including: (1) Complement receptor 1 (Weisman et al., 1990); (2) C1 inhibitor (Buerke et al., 1998); (3) mannose-binding lectin (Busche, Pavlov, Takahashi, & Stahl, 2009; Jordan, Montalto, & Stahl, 2001; Walsh et al., 2005); (4) C5 and C5a (Amsterdam et al., 1995; Vakeva et al., 1998; van der Pals et al., 2010; Zhang et al., 2007); and (5) C3b by cobra venom factor (Gorsuch, Guikema, Fritzinger, Vogel, & Stahl, 2009). Of these therapeutic approaches for targeting the complement cascade, C1 inhibitor therapy and antibodies directed to C5a, have both been tested in clinical studies of AMI, although the results have been mixed (see Section 3).

#### 2.1.2 Damage-associated molecular patterns

**Fig. 2:**
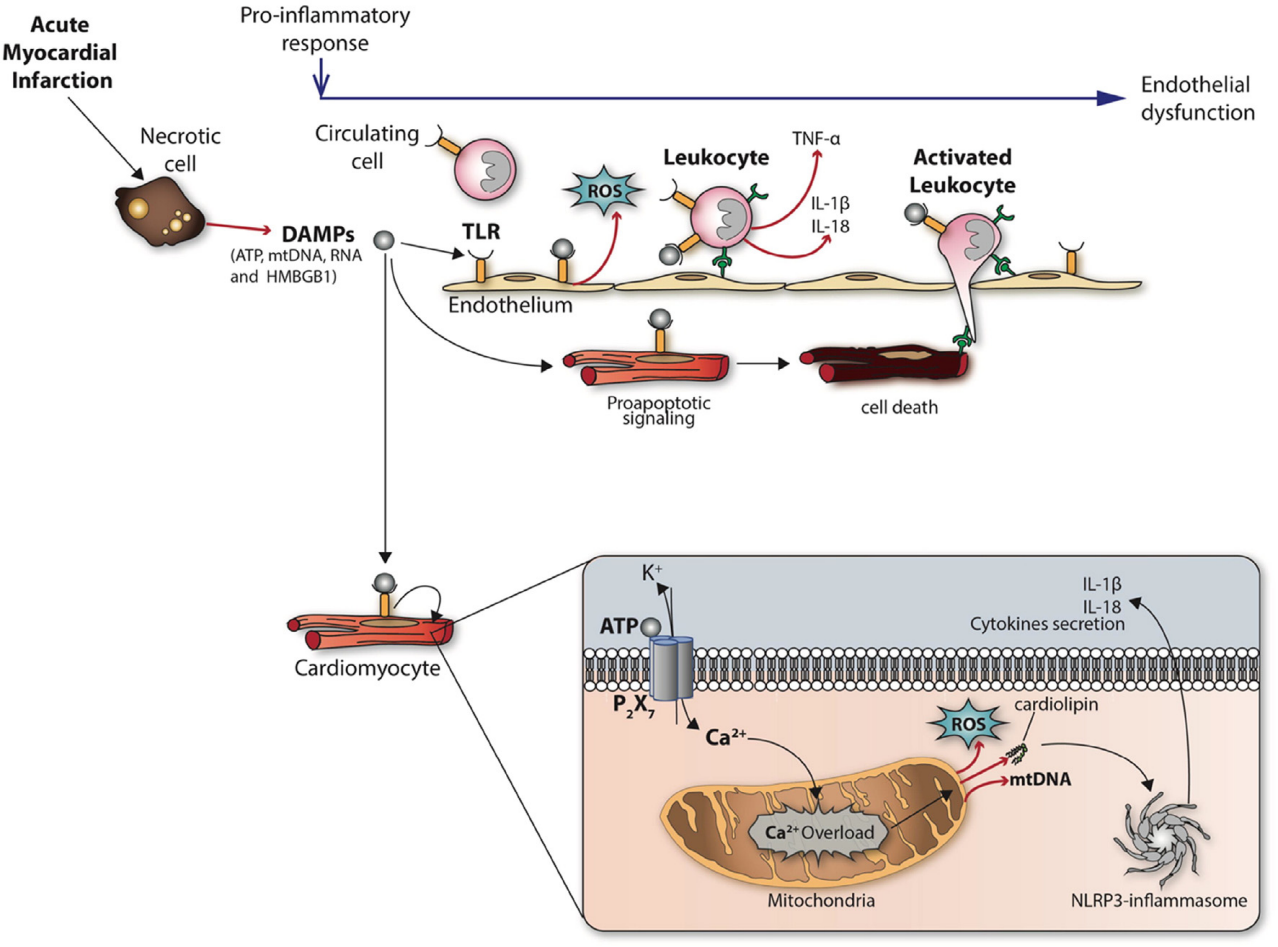
The pro-inflammatory response induced by DAMPs. Following AMI, the release of damage-associated molecular patterns or DAMPs (such as ATP, mtDNA, RNA, and HMBGB1) induce a pro-inflammatory response which mediates cardiomyocyte death through Toll-like receptors (TLRs) and the recruitment of leukocytes into the infarct zone, the release of cytokines, mitochondrial dysfunction (calcium overload and ROS production), and NLRP3-inflammasome formation.

A variety of DAMPs are released from necrotic cardiac resident cells following AMI (such as ATP, mtDNA, RNA and HMBGB1) (Fig. 2). These are known to activate cells of the innate immune system (reviewed in van Hout, Arslan, Pasterkamp, & Hoefer, 2016). The release of ATP can activate the P_2_X_7_ membrane receptor thereby inducing efflux of intracellular potassium, the release of mitochondrial cardiolipin and subsequent NLRP3-inflammasome activation (Iyer et al., 2013; Mariathasan et al., 2006). Extracellular DNA and mtDNA have been reported to induce inflammation by binding to TLR9 (Zhang et al., 2010), and are elevated in patients following AMI (Bliksoen et al., 2012), highlighting a potential anti-inflammatory role for DNAase following AMI. Similarly, the release of extracellular RNA from damaged cardiac resident cells has also been demonstrated to induce a pro-inflammatory response following AMI, through the release of TNF-α and subsequent NF-kB activation (Cabrera-Fuentes et al., 2014; Zernecke & Preissner, 2016) — whether this is mediated by binding to a TLRs is not clear. Again, attenuating circulating levels of extracellular RNA by administering RNAse1 has been shown in experimental small animal studies to reduce MI size (Cabrera-Fuentes et al., 2014; Stieger et al., 2017), and provides a novel therapeutic strategy for reducing MI size.

Following AMI, the nuclear factor, high mobility group box 1 (HMGB1) is released from necrotic cardiac cells, where it has been shown to act on several PRRs including TLR2, TLR4, TLR9 and RAGE to activate NF-kB following AMI (Ding et al., 2013; Park et al., 2004). In support of a pathogenic role for HMGB1, its inhibition following AMI has been reported to be cardioprotective (Andrassy et al., 2008; Hu, Zhang, Jiang, & Hu, 2013). On the other hand, in the post-AMI convalescent phase, HMGB1, at low concentrations, may actually be beneficial and contribute to recovery of LV function, tissue repair, and angiogenesis and regeneration (Kitahara et al., 2008; Takahashi et al., 2008), implicating HMGB1 inhibition at the time of AMI, and HMGB1 activation in the post-MI remodeling phase as therapeutic strategies for reducing MI size and preventing adverse LV remodeling.

#### 2.1.3 Toll-like receptors

In response to DAMPs, TLRs, activate myeloid differentiation primary response gene (MyD)88 and NF-kB nuclear factor-kB (NF-kB), which induce the release of a number of inflammatory mediators including pro-IL-1β and pro-IL-18 (Timmers et al., 2008; van Hout et al., 2016). The TLRs, which are present in circulating and cardiac resident cells, can be divided into those that are mainly present on the cell surface and include TLR1, TLR2, TLR4, TLR5, TLR6 and TLR11, and act as sensors to extracellular DAMPs (such as heat shock proteins [HSPs], HMGB1, fibronectin-end domain A [FN-EDA]); and those which are intracellular such as TLR3, TLR7, TLR8 and TLR9 (which can recognize danger signals such as microbial nucleic acids or self-DNA) (van Hout et al., 2016). Genetic or pharmacological inhibition of either TLR2 or TLR4 have been shown to reduce MI size and prevent adverse LV remodeling following AMI (Arslan et al., 2010; Oyama et al., 2004; Shimamoto et al., 2006; Shishido et al., 2003; Timmers et al., 2008), making these particular TLRs potential therapeutic targets for cardioprotection (see Section 3). Interestingly, a recent experimental study has shown that the environment of the failing and infarcted myocardium was able to polarize resident and transplanted MSCs toward a pro-inflammatory phenotype, and restrict their survival and reparative effects via a TLR4-mediated mechanism (Naftali-Shani et al., 2017).

#### 2.1.4 Inflammasomes

Inflammasomes are large multiple cytoplasmic protein complexes, which form in response to DAMPs released during AMI. They mediate the activation of pro-inflammatory cytokines such as IL-1β and IL-18 from cardiac fibroblasts, and caspase-1 dependent death of cardiomyocytes (termed pyroptosis — a highly inflammatory form of cell death, characterized by both apoptosis and necrosis, reviewed in van Hout et al., 2016). Most inflammasomes typically contain 3 components: (1) a member of the NLRP family proteins (most commonly described in acute myocardial IRI is NLRP3); (2) apoptosis-associated speck-like protein containing a caspase recruitment domain (ASC); and (3) pro-caspase-1 (van Hout et al., 2016). Common upstream mechanisms implicated in NLRP3 inflammasome activation during AMI include extracellular ATP released from injured cells, potassium efflux, lysosomal destabilization, and mitochondrial ROS generation (van Hout et al., 2016).

The release of IL-1β from cardiac fibroblasts, in response to AMI requires two signals: (1) the transcription of pro-IL-1β by the TLR-NF-κB pathway, and (2) the activation of pro-IL-1β to its mature form by the NLRP3 inflammasome. The IL-1β then induces the initial pro-inflammatory response and the release of cytokines/chemokines, which recruit and activate inflammatory cells such as neutrophils and monocytes. Consistent with the role of the NLRP3 inflammasome as a mediator of inflammatory cell death following AMI, both genetic and pharmacological inhibition of its components (caspase 1, IL-1β, ASC, and NLRP3) have been demonstrated to reduce MI size (Coll et al., 2015; Kawaguchi et al., 2011; Marchetti et al., 2014; Mezzaroma et al., 2011; Pomerantz, Reznikov, Harken, & Dinarello, 2001; Sandanger et al., 2013). Interestingly, Toldo et al. (Toldo et al., 2016) found that both MI size and myocardial NLRP3 inflammasome expression in the murine heart increased as the duration of reperfusion was extended over a period of 1, 3 and 24 h, suggesting that the NLRP3 inflammasome may contribute to the phenomenon of ‘late reperfusion injury’, in which reperfusion-induced cell death takes place minutes to hours following reperfusion. Consistent with this finding, the authors found that pharmacological inhibition of the NLRP3 inflammasome (using 16673-34-0, an intermediate of glyburide substrate free of the cyclohexylurea moiety, involved in insulin release), reduced MI size after 24 h reperfusion, when it was administered at the onset of reperfusion or 1 h after reperfusion (but not after 3 h of reperfusion) (Toldo et al., 2016), making it possible to intervene after reperfusion has already taken place in AMI patients treated by PPCI.

#### 2.1.5 Receptor for advanced glycation end-products

The receptor for advanced glycation end-products (RAGE), which is known to act as a receptor for advanced glycation end-products (AGEs) in the setting of diabetes and atherosclerosis, also functions as a PRR for a variety of ligands including HMGB1 and S100/calgranulins in the setting of chronic inflammation. Once it is activated, RAGE facilitates the translocation of NF-kB to the nucleus for transcription of pro-inflammatory cytokines (Shishido et al., 2003).

In the setting of AMI, it has been shown that the administration of recombinant HMGB1 aggravates acute myocardial IRI, suggesting the involvement of RAGE in the post-MI inflammatory response (Andrassy et al., 2008). Mice deficient in RAGE has been shown to be protected against AMI, an effect which was associated with less accumulation of inflammatory cells into the MI zone (Volz et al., 2012). Furthermore, macrophages and cardiac fibroblasts subjected to simulated ischemia demonstrated increased S100A8/A9-mediated RAGE activation (Volz et al., 2012). Accordingly, combined blockade of RAGE signaling by siRNA and soluble RAGE synergistically protected from cardiac damage after MI (Ku et al., 2015).

#### 2.1.6 Cytokines

Following AMI, a number of pro-inflammatory cytokines are secreted by cardiac resident cells and circulating inflammatory cells. They play a critical role in amplifying the pro-inflammatory response to acute myocardial IRI by mediating the recruitment of inflammatory cells into the MI zone. The major cytokine mediating the pro-inflammatory response following AMI is interleukin-1 (IL-1). In AMI experimental models, IL-1α is released by damaged cardiomyocytes, whereas IL-1β is upregulated after infarction. Both genetic and pharmacological inhibition of IL-1 has been shown to reduce MI size and prevent adverse LV remodeling (Abbate et al., 2008; Bujak et al., 2008). Small clinical trials have tested the effectiveness of IL-1 inhibition in patients with AMI (see Section 3).

IL-6 has been reported to play both pro- and anti-inflammatory roles, and is released following acute myocardial IRI (Gwechenberger et al., 1999). Elevated circulating IL-6 levels have been shown to be associated with acute coronary syndromes (Empana et al., 2010). Mendelian randomization studies have demonstrated that genetic polymorphisms in the IL-6 receptor signaling pathway resulted in lower plasma levels of high-sensitivity C-reactive protein (hs-CRP) and reduced cardiovascular risk (Swerdlow et al., 2012), suggesting that IL-6 may be detrimental in patients with coronary artery disease. However, genetic or pharmacological modulation of IL-6 in the experimental setting of AMI have produced mixed results. Mice deficient in IL-6 have been reported to sustain either similar MI size (Fuchs et al., 2003; Kaminski et al., 2009) or smaller MI size (Jong et al., 2016) when compared to wild-type mice. Similarly, it has been shown that administering the IL-6 receptor antibody, MR16-1, prior to reperfusion and weekly for 1 month, actually worsened adverse LV remodeling in a reperfused murine AMI model (Hartman et al., 2016), suggesting a beneficial role for IL-6 in this setting. This illustrates the challenges in targeting the different components of the inflammatory response to AMI with respect to the discordant effects which can arise depending on the timing of the intervention. Despite the mixed experimental data, clinical studies have been performed targeting IL-6 in the clinical setting (see Section 3).

#### 2.1.7 Chemokines

The chemokines are a large family of chemoattractant cytokines which are secreted in response to pro-inflammatory cytokines — these play an important role in selectively recruiting monocytes, neutrophils, and lymphocytes. The CC chemokine, monocyte chemoattractant protein-1 (MCP-1)/chemokine ligand 2 (CCL2), is rapidly upregulated in the infarcted heart, and acts as a potent chemoattractant to mononuclear cells (Kumar et al., 1997). Genetic ablation of MCP-1 or its receptor, chemokine receptor 2 (CCR2), reduced recruitment of pro-inflammatory monocytes, and decreased cytokine expression in the infarct zone, and prevented adverse remodeling following AMI (Dewald et al., 2005). Injection of anti-MCP-1 into the skeletal muscle one month following a non-reperfused MI prevented adverse LV remodeling and reduced mortality (Hayashidani et al., 2003). The CC chemokine, chemokine ligand 5 (CCL5), also plays a critical role as a chemoattractant for neutrophils and macrophages following AMI. It has been shown that treatment with an anti-mouse CCL5 monoclonal Ab following a non-reperfused infarct reduced MI size, decreased circulating levels of chemokines, attenuated reduction of neutrophil and macrophage infiltration within the infarcted myocardium, prevented adverse LV remodeling and reduced mortality (Montecucco et al., 2012). However, it has also been shown that mice deficient in chemokine receptor 5 (CCR5) (Dobaczewski, Xia, Bujak, Gonzalez-Quesada, & Frangogiannis, 2010) exhibited enhanced myocardial inflammation, enhanced matrix metalloproteinase expression, and worsened adverse LV remodeling following reperfused MI, findings which were associated with impaired recruitment of anti-inflammatory CCR5^+^ foxp3^+^ regulatory T cells (Tregs). More recently, the role of the CC chemokine receptor 9 (CCR9) (mainly expressed in lymphocytes, dendritic cells and monocytes/macrophages) has been investigated in the setting of AMI (Huang et al., 2016). Mice deficient in CCR9 had less mortality following AMI, smaller MI size and less adverse LV remodeling, effects which were associated with attenuated inflammation and decreased inflammatory signaling through NF-κB and MAPK pathways (Huang et al., 2016). Finally, pharmacological inhibition of the neutrophil attracting CXC chemokine during myocardial ischemia reduced MI size by preventing CXC chemokine-induced neutrophil recruitment and reactive oxygen species production following AMI (Montecucco et al., 2010).

#### 2.1.8 Endothelial cells

Following AMI, DAMPs released by dying cardiomyocytes induce activation of endothelial cells within the heart — this is characterized by increased production of ROS and pro-inflammatory cytokines, and enhanced expression of adhesion molecules which mediate the binding of Leukocytes and platelets (Prabhu & Frangogiannis, 2016). Intercellular tight junctions between endothelial cells become compromised following acute myocardial IRI resulting in leaky coronary endothelium and increased coronary microvascular permeability dysfunction, key determinants of reperfused-induced cardiomyocyte death following AMI.

#### 2.1.9 Neutrophils

Neutrophils normally provide the first line of defense against invading microorganisms and tissue injury (reviewed in Prabhu & Frangogiannis, 2016). Following AMI, polymorphonuclear leukocytes (PMNs) in the bone marrow mobilize into the blood, and are the first inflammatory cells to arrive at the injured myocardium, being present within hours following AMI, peaking at days 1–3, and starting to decline at day 5. Neutrophils tend to target the border zone of the MI and their accumulation is accentuated at reperfusion. The PMNs are recruited into the injured myocardium in response to a high concentration of chemotactic factors, such as macrophage inflammatory protein-2α (MIP-2α, CXCL2, GRO β), leukotriene B4 (LTB4), cytokine-induced neutrophil chemoattractant 1 (CINC-1, CXCL1, GRO α, KC), interleukin 8 (IL-8, CXCL8), and complement 5a. Here, they leave the circulation and infiltrate the injured myocardium across the endothelium of post-capillary venules in 3 sequential steps (a process termed extravasation):

(1) The PMNs first adhere to and roll on endothelial cells by binding to P-selectin, E-selectin, intercellular adhesion molecules (ICAMs), and vascular cell adhesion molecules expressed on activated endothelial cells.
(2) Firm adhesion then occurs by interaction of the integrins, α_L_β_2_ and α_M_β, present on PMNs with their ligands ICAM-1 and ICAM-2 on endothelial cells.
(3) Finally, trans-endothelial migration of the PMNs across the endothelial cells is also mediated by the integrins α_L_β_2_ and α_M_β_2_, ICAM-1, and ICAM-2.

Once in the injured myocardium, the PMNs play multiple roles including phagocytosis of cellular debris, degradation of extracellular matrix through the release of granules containing matrix metalloproteinases (MMPs), generating ROS, and secreting factors that are chemotactic to monocytes. Excessive PMN infiltration and/or their delayed removal may exacerbate myocardial injury by prolonging the pro-inflammatory response.

Interestingly, Ma et al. (2016) have recently suggested that infiltrating neutrophils may be polarized following AMI, opening up opportunities for therapeutic modulation of neutrophil polarization as a strategy for preventing inflammation and cardioprotection. They found that neutrophils harvested from myocardium at day 1 following AMI had high expression of pro-inflammatory markers and were polarized by TLR4 activation (termed N1 neutrophils and induced by lipopolysaccharide and interferon-γ), whereas those collected from the heart at days 5–7 post-MI, were anti-inflammatory (termed N2 neutrophils and induced by interleukin-4).

Loss of the CD11/CD18-integrin receptor (which allows neutrophils to bind to and traverse the endothelium) has been demonstrated to reduce acute MI size in small and large animal AMI models (Arai et al., 1996; Aversano, Zhou, Nedelman, Nakada, & Weisman, 1995; Lefer et al., 1993; Tanaka et al., 1993), making it a therapeutic target, a strategy which has been tested in the clinical setting of AMI (see Section 3).

#### 2.1.10 Monocytes and macrophages

Monocytes produced in the bone marrow and spleen enter the blood after AMI and are recruited to the injured myocardium in 2 phases. The first phase is dominated by inflammatory Ly-6c^high^ monocytes (peaking day 3–4 post MI), and the second phase by anti-inflammatory Ly-6c^low^ monocytes (peaking day about day 7 post-MI). The infiltrating monocytes then differentiate into M1 macrophages responsible for clearing cell debris from the MI zone. Subsequently, cytokines, chemokines and growth factors secreted by M1 macrophages influence the reparative phase co-ordinated by M2 macrophages. However, the prolonged presence of M1 macrophages can extend the pro-inflammatory phase and cause expansion of the infarcted area, thereby delaying the reparative phase and formation of scar tissue mediated by M2 macrophages and exacerbating adverse LV remodeling. As such, therapeutic modulation of macrophage polarization may provide a novel treatment strategy for reducing MI size and preventing adverse LV remodeling (reviewed in ter Horst et al., 2015). It has been shown in experimental studies that targeting pro-inflammatory monocytes or M1 to suppress the pro-inflammatory phase post-MI, is cardioprotective (Courties et al., 2014; Harel-Adar et al., 2011; Leuschner et al., 2011). Conversely, promoting M2 macrophage polarization has been demonstrated to facilitate the resolution of inflammation and prevent adverse LV remodeling following AMI (Weirather et al., 2014; Zhou et al., 2015).

#### 2.1.11 T and B lymphocytes

Experimental small and large animal models have demonstrated infiltration of both T and B lymphocytes into the MI zone following AMI (Frangogiannis et al., 2000; Yan et al., 2013; Zouggari et al., 2013). It has been shown that circulating cytotoxic T (CD8) lymphocytes increase one week following AMI, and when harvested from rats subjected to AMI, they were found to exert a cytotoxic effect on healthy neonatal cardiomyocytes (Varda-Bloom et al., 2000). Whether infiltrating T cells are able to exacerbate acute ischemic injury *in vivo* is not clear. The role of T cells in mediating acute myocardial IRI has recently been investigated in ST-segment elevation myocardial infarction (STEMI) patients treated by PPCI. It was demonstrated that at 90 min after reperfusion there was a reduction in circulating T cells (predominantly CD8^+^), most pronounced in patients with microvascular obstruction on cardiac MRI, an effect which coincided with a peak elevation in the expression of the fractalkine receptor CX3CR1 (Boag et al., 2015). In contrast, another study has shown that circulating levels of CD8 T cells in AMI patients were associated with short-term (6 months) cardiovascular mortality, suggesting that they might contribute to acute coronary events via their pro-inflammatory cytotoxic effects.

It has been shown that following AMI, mature B lymphocytes infiltrate into the MI zone (peaking at day 5 post-AMI), and augment the pro-inflammatory response by secreting the chemokine CCL7, which in turn induces mobilization from the bone marrow of pro-inflammatory Ly6C^hi^ monocytes (Zouggari et al., 2013). Genetic depletion of B lymphocytes using specific antibodies to CD20 or against B-cell activating factor (BAFF) (a B lymphocyte survival factor) was shown to attenuate systemic inflammation (measured by systemic IL-1β, TNF and IL-18), reduce MI size and improve LV function by day 14 post-AMI (Zouggari et al., 2013), implicating B cells as a new therapeutic target for preventing inflammation post-AMI.

#### 2.1.12 Cardiac fibroblasts

In addition to their contribution to scar formation and matrix remodeling, cardiac fibroblasts play an important role in the inflammatory response to AMI and subsequent LV remodeling (reviewed in Shinde & Frangogiannis, 2014). They are known to be activated by DAMPs released by damaged cardiomyocytes. In the first 24–72 h following AMI, the cardiac fibroblasts act as pro-inflammatory mediators, activating the inflammasome and producing cytokines, chemokines and exhibiting matrix-degrading properties, thereby helping to clear the wound of dead cells and remove matrix debris — this process is facilitated by specific pro-inflammatory cytokines (such as interleukin-1 which inhibits α-SMA expression by fibroblasts), which delay the transformation of fibroblasts into myofibroblasts.

### 2.2 The anti-inflammatory reparative phase following AMI

**Fig. 3:**
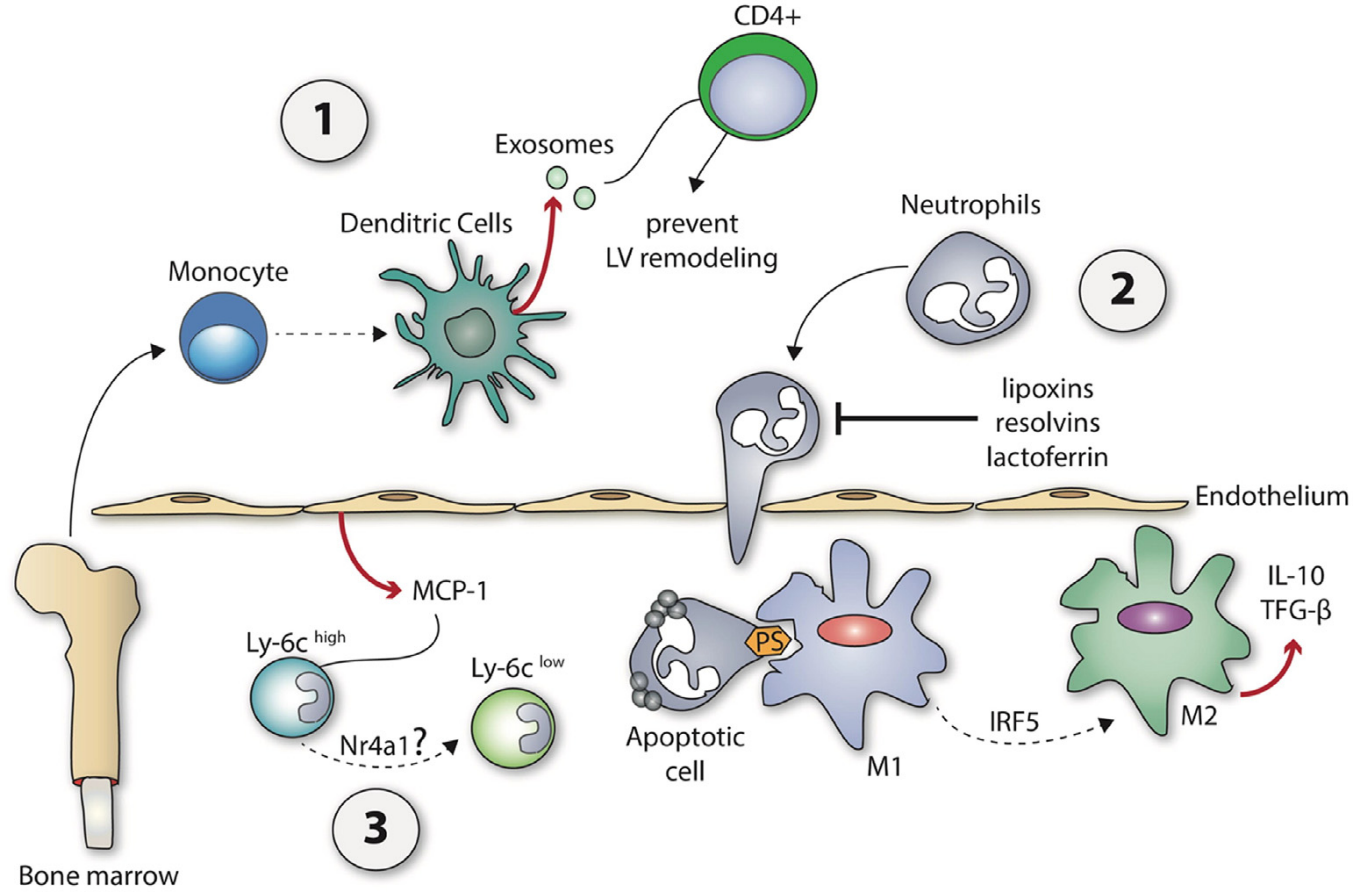
The anti-inflammatory reparative phase following acute myocardial infarction. Following the pro-inflammatory response of AMI, the anti-inflammatory reparative phase allows the resolution of inflammation. (1) Bone marrow and circulating monocytes are reported to differentiate into dendritic cells that prevent LV remodeling by the exosome activation of CD4^+^ leukocytes. (2) PS expression of apoptotic neutrophils induces M2 macrophage polarization and the secretion of anti-inflammatory and pro-fibrotic cytokines such as IL-10 and TGF-β that suppress inflammation and promote tissue repair. (3) A switch from pro-inflammatory Ly6C^hi^ monocytes and M1 macrophages localized at the MI zone in response to increased myocardial CCL-2/MCP-1 expression during the initial pro-inflammatory phase to anti-inflammatory Ly6C^low^ monocytes and M2 macrophages, possibly mediated by Nr4a1 and in the case of macrophages mediated by IRF5.

The anti-inflammatory reparative phase (days 4–7) following AMI is orchestrated by suppression, resolution and containment of the initial pro-inflammatory response (Fig. 3). This is driven by the activation of specific endogenous inhibitory pathways that suppress inflammation, and dynamic changes in the roles of infiltrating leukocytes within the MI zone.

#### 2.2.1 Neutrophils

Apoptosis of neutrophils and their subsequent clearing from the MI zone is a hallmark of inflammation resolution and the reparative phase. It is an active process that requires the recruitment of a number of inhibitory pathway cascades (Mantovani, Cassatella, Costantini, & Jaillon, 2011; Serhan, Chiang, & Van Dyke, 2008). The expression of phosphatidylserine (PS) facilitates the ingestion of apoptotic neutrophils by macrophages, resulting in M2 macrophage polarization and the secretion of anti-inflammatory and pro-fibrotic cytokines such as IL-10 and TGF-β that suppress inflammation and promote tissue repair (Ortega-Gomez, Perretti, & Soehnlein, 2013). Furthermore, the release of anti-inflammatory mediators such as pro-resolving lipid mediators (e.g., lipoxins and resolvins), annexin A1, and lactoferrin, act to prevent neutrophil transmigration and entry, and promote neutrophil apoptosis and the phagocytic uptake of apoptotic neutrophils by macrophages (Ortega-Gomez et al., 2013).

#### 2.2.2 Monocytes and macrophages

It has been demonstrated that dynamic changes in the polarization of monocytes and macrophages from proteolytic and pro-inflammatory Ly6C^hi^ and M1 phenotypes (peak day 3–4 following AMI), respectively, to anti-inflammatory (e.g., IL-10, TGF-β, and vascular endothelial growth factor) Ly6C^lo^ and M2 phenotypes (peak day 7 following AMI) are critical to the reparative phase following AMI (Nahrendorf et al., 2007; Nahrendorf, Pittet, & Swirski, 2010). The current paradigm suggests that pro-inflammatory Ly6C^hi^ monocytes infiltrate the MI zone in response to increased myocardial CCL-2/MCP-1 expression during the initial pro-inflammatory phase, and then the Ly6C^hi^ monocytes switch their phenotype to anti-inflammatory Ly6C^low^ monocytes, in the reparative phase. The factors mediating this transition in monocyte phenotype remain unclear although Nr4a1 has been suggested to play a role (Hanna et al., 2011; Hilgendorf et al., 2014). A number of factors have been implicated as mediating the polarization changes from M1 to M2 macrophages following AMI, such as interferon regulatory factor 5 (IRF5) (Courties et al., 2014). Pharmacological modulation of monocyte and macrophage polarization to their anti-inflammatory phenotypes, may therefore provide a therapeutic strategy for augmenting the reparative phase following AMI.

#### 2.2.3 Dendritic cells

Following tissue injury, bone marrow and splenic precursors and circulating monocytes are reported to differentiate into dendritic cells — these exert various influences on the immune system at the inflammatory site, such as priming of antigen-specific immune responses, induction of tolerance, and chronic inflammation. It has been shown that following an AMI, dendritic cells migrate from the bone-marrow into the ischemic zone accumulating in the MI and border zones (peaking at 7 days post-MI). Anzai et al. (2012) demonstrated that depletion of dendritic cells from the bone marrow in mice extended the pro-inflammatory phase following AMI to 7 days as evidenced by: (1) sustained elevation of pro-inflammatory cytokines such as IL-1β, IL-18, TNF-α, and CCL2 and inhibition of anti-inflammatory cytokines such as IL-10 and CX3CL1; (2) increased pro-inflammatory Ly6C^high^ monocyte but decreased anti-inflammatory Ly6C^low^ monocyte infiltration into the MI zone; (3) enhanced infiltration of pro-inflammatory M1 macrophages and attenuated recruitment of anti-inflammatory M2 macrophages into the MI zone. These pro-inflammatory effects were associated with worse adverse LV remodeling following AMI as evidenced by accelerated cardiac dilatation and deterioration of LV function. These results suggested a protective role of dendritic cells in the inflammatory response to AMI and subsequent LV remodeling. Interestingly, a recently published study has found that exosomes produced by dendritic cells may recruit CD4^+^ helper T cells into the MI zone and help prevent adverse LV remodeling post-AMI (Liu et al., 2016).

#### 2.2.4 Regulatory T lymphocytes (Tregs)

Emerging evidence suggests that CD4^+^CD25^+^FOXP3^+^ regulatory T lymphocytes (Tregs) may play an anti-inflammatory (immunosuppressive) role in the setting of AMI through the secretion of anti-inflammatory cytokines (such as TGF-β and IL-10), and cell-contact-dependent interaction with other cell types (reviewed in Meng et al., 2016; Wang et al., 2016). Tregs constitute a specific subset of T lymphocytes with immunosuppressive capacity, and make up 5–10% of circulating CD4^+^ T lymphocytes under physiological conditions (Wang & Alexander, 2013).

Following AMI, the infiltration of Tregs into the MI zone has been demonstrated to have several beneficial effects in terms of reducing MI size and preventing adverse LV remodeling. Experimental studies have demonstrated that mice deficient in Tregs experience larger MI size and more adverse LV remodeling (Hofmann et al., 2012) whereas the infusion of this cell population reduced MI size and preserved myocardial function in murine AMI models (Matsumoto et al., 2011). The protective effects of Tregs following AMI has been attributed to the following:

1. Inhibiting the recruitment of inflammatory cells (neutrophils, monocytes, CD4^+^ T lymphocytes etc.) and suppress local expression of pro-inflammatory cytokines such as TNF-α and IL-1β (Hofmann et al., 2012; Tang et al., 2012);
2. Promoting polarization of macrophages to an anti-inflammatory M2 phenotype, and inhibiting polarization to a pro-inflammatory M1 phenotype (Weirather et al., 2014);
3. Inhibiting the transdifferentiation of fibroblasts into myofibroblasts and downregulation of pro-fibrotic MMPs, thereby preventing adverse LV remodeling (Saxena et al., 2014);
4. Preventing cardiomyocyte apoptosis (Tang et al., 2012);

Modulation of Tregs by pharmacological cardioprotective interventions such as rosuvastatin (Ke, Fang, Fan, Chen, & Chen, 2013) and FTY720 (an analogue of sphingosine-1-phosphate) (Wang et al., 2014) has been reported in experimental AMI models. Whether Tregs mediate the cardioprotection induced by endogenous strategies such as ischemic preconditioning and postconditioning is not known and remains to be tested.

Interestingly, patients presenting with an AMI have been demonstrated to have decreased levels of circulating Tregs, compared with control patients (Mor, Luboshits, Planer, Keren, & George, 2006; Sardella et al., 2007). Furthermore, it has been shown that low levels of Tregs at baseline are associated with a higher risk for future AMI (Wigren et al., 2012). The reduction in Tregs following AMI has been attributed to a number of factors including: (1) accumulation of Tregs in MI zone (Saxena et al., 2014); (2) attenuated production of Tregs by thymus (Zhang et al., 2012); and (3) increased apoptosis of Tregs (Zhang et al., 2012). These clinical studies, suggest a protective role of Tregs in MI, and they may therefore, present an important therapeutic target for reducing MI size and preventing adverse remodeling in AMI patients. Preliminary clinical studies have reported ex vivo expanded human TREG cells were safe and effective in the prevention and treatment of graft-versus-host disease or type 1 diabetes mellitus (Brunstein et al., 2011; Marek-Trzonkowska et al., 2014; Trzonkowski et al., 2009). Whether this therapeutic approach can be applied to prevent adverse LV remodeling following AMI remains to be tested.

#### 2.2.5 Other T lymphocyte subpopulations

Other subpopulations of T lymphocytes may also contribute to the reparative phase following AMI. CD4^+^ helper T cells have been shown to be activated following AMI (probably in response to released cardiac autoantigens). They have been demonstrated to contribute to resolution of inflammation and wound healing with collagen matrix formation and scar formation to prevent adverse LV remodeling (Hofmann et al., 2012). Invariant natural killer lymphocytes have been reported to be activated following AMI, and reduce leukocyte infiltration, lessen myocardial injury, and prevent adverse LV remodeling, beneficial effects which were shown in part, to be due expression of anti-inflammatory cytokines such as IL-10 (Homma et al., 2013; Sobirin et al., 2012).

#### 2.2.6 Cardiac fibroblasts

During the proliferative phase of MI healing, the loss of pro-inflammatory signals such as IL-1β and Interferon-γ-inducible Protein (IP)-10, allows cardiac fibroblasts within the MI border zone to transdifferentiate into myofibroblasts (reviewed in Shinde & Frangogiannis, 2014). These express the contractile protein, α-smooth muscle actin (α-SMA), and exhibit an extensive endoplasmic reticulum, and are capable of secreting large amounts of matrix proteins. Factors contributing to the trans-differentiation of cardiac fibroblasts into α-SMA myelofibroblasts include TGF-β signaling, modulation of the matrix environment (expression of ED-A fibronectin) and deposition of non-fibrillar collagens (such as collagen VI), expression of proteoglycans by cardiac fibroblasts, and mechano-sensitive signaling.

The formation of a mature scar signifies the end of the proliferative phase and is associated with matrix cross-linking and the progressive clearance of myelofibroblasts by apoptosis. The mechanisms underlying this process are unclear but may involve the withdrawal of growth factors, specific inhibition of the TGF-β-driven fibrotic response, and alterations in the composition of the matrix such as the small leucine-rich proteoglycan biglycan.

In the non-infarcted remote myocardium, cardiac fibroblasts may remain chronically activated in response to pressure overload inducing early activation of matrix synthetic pathways, associated with cardiac fibrosis and diastolic dysfunction, a process which is followed by activation of matrix-degrading signals, chamber dilatation and systolic dysfunction. In contrast, volume overload is primarily associated with matrix loss and cardiac dilation. The mechanisms which regulate these differential matrix response to volume and pressure overload remain unclear, although stretch and humoral triggers of remodeling concurrent with loss of contractile tissue and heart failure may be involved (Burchfield, Xie, & Hill, 2013; Dorn, 2009). Elucidation of these regulatory pathways may identify novel therapeutic targets for modulating the fibrotic response to AMI and help to prevent post-MI adverse remodeling.

### 2.3 The contribution of persistent or chronic inflammation to post-MI adverse LV remodeling

Following AMI, the LV undergoes geometric and functional changes, with hypertrophy of the non-infarcted segments and dilatation/thinning of the infarcted segments resulting in reduced LV ejection fraction — a process termed adverse LV remodeling, and the occurrence of which is associated with worse clinical outcomes. There is preliminary evidence that an excessive, persistent and expanded pro-inflammatory response following AMI may worsen post-MI adverse LV remodeling through the following processes: activating proteases (Dobaczewski et al., 2010); increasing cytokine expression which may induce cardiomyocyte apoptosis and suppress contractility; increasing matrix deposition which may result in a stiffer ventricle and causes diastolic dysfunction (Bujak et al., 2008); and the activation of cardiac fibroblasts in the infarct border zone which may expand fibrosis into viable tissue. Experimental studies have demonstrated that myocardial expression levels of pro-inflammatory cytokines (IL-6, TNF-α, and IL-1β) at 8 and 20 weeks after AMI in a rat model, were significantly associated with left ventricular end-diastolic volume (LVEDV) (Ono, Matsumori, Shioi, Furukawa, & Sasayama, 1998). Similarly in STEMI patients, IL-1β levels measured pre-, 2, and 7 days post-PPCI were able to predict indexed left ventricular end-systolic volume (LVESV) and LVEDV values measured by CMR at 1 year (Orn et al., 2012). Therefore, modulating the persistent or chronic inflammatory response to AMI, may provide therapeutic targets for preventing adverse LV remodeling following AMI.

## 3 Therapeutic targeting of inflammation following MI

Given the detrimental effects of an excessive and persistent pro-inflammatory response to AMI, and the beneficial healing effects of the anti-inflammatory reparative phase which follows, a potential therapeutic strategy for limiting MI size and preventing adverse LV remodeling, is to attenuate the initial pro-inflammatory response, and upregulate the subsequent anti-inflammatory reparative response. Separate to the role of inflammation in AMI, it has been postulated that persistent or chronic inflammation following an AMI, may result in adverse LV remodeling, providing an additional therapeutic target for preventing post-MI heart failure (Westman et al., 2016). A number of therapeutic approaches aimed at targeting the pro-inflammatory response following AMI have been investigated, many of which have, unfortunately, failed to demonstrate any benefit on reducing MI size or improving clinical outcomes (see Table 1 for a summary of the major clinical studies), further details of which will be discussed in more detail in Section 3.6. Therefore, novel therapeutic strategies are required to target the inflammatory response to AMI, and some of these are highlighted in this section.

**Table 1:** Major clinical studies investigating an anti-inflammatory therapeutic strategy to protect the myocardium against acute ischemia/reperfusion injury.

Target	Clinical study	Patient population	Treatment	Outcome	Mechanism
Neutrophils	Baran et al. (2001) LIMIT AMI	394 STEMI thrombolysed <12 h	IV rhuMAb CD18 0.5 mg/kg or 2.0 mg/kg prior to thrombolysis	No effects on coronary blood flow, MI size (SPECT), or ST-segment resolution	rhuMAb CD18 is a monoclonal antibody to the CD18 subunit of the β2 integrin adhesion receptors to prevent neutrophil adhesion
Neutrophils	Faxon, Gibbons, Chronos, Gurbel, and Sheehan (2002) HALT MI	420 STEMI PPCI <6 h Per-PPCI TIMI ≤1	IV Hu23F2G 0.3 mg/kg or 1.0 mg/kg prior to PPCI	No effects on MI size (SPECT), corrected TIMI frame count or clinical events at 30 days	Hu23F2G (LeukArrest), a humanized MAb to the neutrophils integrin receptor CD11/CD18
Complement cascade C1	de Zwaan et al. (2002)	22 STEMI thrombolysed <12 h	IV C1-inhibitor 48 h infusion initiated 6 h after reperfusion	Reduction in MI size (CK-MB or Trop T) but small study	Cetor is a monoclonal antibody to human C1-inhibitor which inhibits activation of the complement cascade.
Complement cascade C1	Thielmann et al. (2006)	57 STEMI emergency CAG <12 h	IV C1-inhibitor 6 h infusion initiated 10 min prior to reperfusion (unclamping of aorta)	Reduction in peri-operative myocardial injury size (Trop I)	Berinert is a monoclonal antibody to human C1-inhibitor which inhibits activation of the complement cascade.
Complement cascade C1	Fattouch et al. (2007)	80 STEMI emergency CAG <12 h	IV C1-inhibitor 3 h infusion initiated 10 min prior to reperfusion (unclamping of aorta)	Reduction in peri-operative myocardial injury size (Trop I)	A monoclonal antibody to human C1-inhibitor which inhibits activation of the complement cascade.
Complement cascade C5	Mahaffey et al. (2003) COMPLY	943 STEMI thrombolysed <6 h	IV Pexelizumab 2.0-mg/kg bolus, or 2.0-mg/kg bolus plus 0.05 mg/kg/h for 20 h prior to or soon after start of thrombolysis	No effects on MI size (CK-MB or Trop I) or clinical events	Pexelizumab, is an Anti-C5 Complement Antibody which inhibits activation of the complement cascade.
Complement cascade C5	Granger et al. (2003) COMMA	960 STEMI PPCI <6 h	IV Pexelizumab 2.0-mg/kg bolus plus 0.05 mg/kg/h for 20 h prior to PPCI	No effects on MI size (CK-MB). However, reduction in mortality at 90 days.	Pexelizumab, is an Anti-C5 Complement Antibody which inhibits activation of the complement cascade.
Complement cascade C5	Verrier et al. (2004) PRIMO-CABG	3099 CABG ±valve	IV Pexelizumab 2.0-mg/kg bolus plus 0.05 mg/kg/h for 20 h prior to or soon after start of thrombolysis	No effects on peri-operative MI size (CK-MB or Trop I) or clinical events	Pexelizumab, is an Anti-C5 Complement Antibody which inhibits activation of the complement cascade.
Complement cascade C5	Armstrong et al. (2007) APEX MI	5745 STEMI PPCI Ant/Inferolat <6 h	IV Pexelizumab 2.0-mg/kg bolus plus 0.05 mg/kg/h for 24 h prior to PPCI	No effects on mortality at 30 days.	Pexelizumab, is an Anti-C5 Complement Antibody which inhibits activation of the complement cascade.
Fibrin	Atar et al. (2009) FIRE	234 STEMI PPCI	IV FX-06400 mg in 2 divided doses at time of PPCI	49% reduction in 7 day AUC hsCRP non-significant 21% (*P* = .21) reduction in MI size (LGE MRI on day 5). No difference in 48 h troponin.	FX06 is a naturally occurring peptide fragment of fibrin which prevents binding to an endothelial specific molecule, VE-cadherin, thereby reducing plasma leakage into tissues and acting as an anti-inflammatory agent.
IL-1	Abbate et al. (2010) VCU-ART	10 STEMI PPCI	Subcutaneous IL-1receptor antagonist (IL-1ra, 100 mg) or placebo daily for 14 days.	Smaller increase in index LVESV at 10–14 weeks assessed by MRI.	Anakinra (Kineret™ from Amgen) is a humanized anti-IL-1R antibody.
IL-1	Abbate et al. (2013) VCU-ART2	25 STEMI PPCI pooled analysis	Subcutaneous IL-1receptor antagonist (IL-1ra) or placebo for 14 days.	Failed to show a statistically significant effect on indexed LVESV, LVEDV or LVEF.	Anakinra (Kineret™ from Amgen) is a humanized anti-IL-1R antibody.
IL-1	Morton et al. (2015) MRC-ILA Heart Study	182 NSTEMI undergoing PCI	Subcutaneous IL-1receptor antagonist (IL-1ra) or placebo for 14 days.	49% reduction in 7 day AUC hsCRP. However, there was an increase in MACE (death, stroke, and new MI).	Anakinra (Kineret™ from Amgen) is a humanized anti-IL-1R antibody.
P-selectin	Tardif et al. (2013) SELECT-ACS	322 NSTEMI undergoing PCI	IV Inclacumab (20 mg/kg) initiated prior to angiography for 1 h	24% and 34% reduction in peak Trop I at 16 and 24 h post-PCI (borderline significant)	Inclacumab is a humanized antibody that inhibits P-selectin, an adhesion molecule involved in interactions between endothelial cells, platelets, and leukocytes.
IL-6	Kleveland et al. (2016)	117 NSTEMI undergoing PCI	IV Tocilizumab (20 mg/ml) initiated prior to angiography for 1 h	52% reduction in median AUC hsCRP and 22% reduction in median AUC hsTropT	Tocilizumab is a humanized anti-IL-6R antibody that binds to both membrane-bound and soluble (s) IL-6R

### 3.1 Corticosteroids and non-steroidal anti-inflammatory drugs (NSAIDS)

Early experimental studies using non-specific anti-inflammatory agents such as corticosteroids and NSAIDs showed a reduction in MI size (Kirlin et al., 1982; Libby, Maroko, Bloor, Sobel, & Braunwald, 1973). Although this therapeutic strategy was initially associated with adverse effects such as impaired myocardial repair, myocardial thinning and cardiac rupture in the clinical setting (Hammerman, Kloner, Hale, Schoen, & Braunwald, 1983), later studies showed that prolonged corticosteroid therapy-induced aneurysm formation was related to inhibition of the reparative phase, as well as the intended acute pro-inflammatory phase (Garcia, Go, & Villarreal, 2007; Jugdutt, Khan, Jugdutt, & Blinston, 1995).

### 3.2 Complement cascade inhibition

A number of experimental studies have reported that C1 and C5/C5a inhibition of the complement cascade following AMI can reduce MI size. However, several small and large clinical trials have investigated the effect of Pexelizumab, an Anti-C5 Complement Antibody, but have failed to find any beneficial effects on clinical outcomes in either reperfused STEMI (Mahaffey et al., 2003) or CABG patients (see Table 1 for details). Preliminary experience with intravenous C1 inhibition infusion therapy has reported reduced myocardial injury in STEMI patients treated by thrombolysis or emergency CABG surgery (see Table 1 for details). Whether C1 inhibition therapy would be effective in STEMI patients treated by PPCI is not known.

### 3.3 Neutrophils

Loss of the CD11/CD18-integrin receptor (which allows neutrophils to bind to and traverse the endothelium) has been demonstrated to reduce acute MI size in small and large animal AMI models (Arai et al., 1996; Aversano et al., 1995; Lefer et al., 1993; Tanaka et al., 1993). However, several clinical trials have demonstrated that the administration of monoclonal antibodies directed to the CD11/CD18-integrin receptor failed to reduce acute MI size and improve short-term clinical outcomes at 30 days in STEMI patients treated by reperfusion therapy (see Table 1) (Baran et al., 2001; Faxon et al., 2002).

### 3.4 P-selectin Ab

In the Select-ACS trial, Inclacumab, a recombinant monoclonal antibody against P-selectin, administered prior to PCI in NSTEMI patients, has been found to reduce peri-procedural myocardial injury, as evidenced by less Troponin I and CK-MB (Tardif et al., 2013). Whether this therapeutic approach would be effect in reducing MI size and prevent adverse LV remodeling in STEMI patients undergoing PPCI is not known and remains to be tested.

### 3.5 Therapeutic targeting of inflammatory cytokines

Inflammatory cytokines constitute intriguing therapeutic targets because of their pleiotropic effects on immune responses. Despite the promising results shown in animal models, however, clinical studies of therapies targeting inflammatory cytokines and chemokines have produced mixed results.

#### 3.5.1 IL-1β

Two small clinical trials (VCU-ART 1 and 2) have shown that 2 weeks daily subcutaneous administration of the IL-1 inhibitor, Anakinra, was safe and suppressed serum CRP levels in the post-MI period, although the effects on preventing adverse LV remodeling were mixed (see Table 1) (Abbate et al., 2010, Abbate et al., 2013). The ongoing VCU-ART3 study is currently investigating the effect of Anakinra on serum CRP levels and post-MI LV remodeling in a larger population of 99 STEMI patients (ClinicalTrials.gov Identifier NCT01950299). In NSTEMI patients undergoing PCI, serum hs-CRP levels were again suppressed, although there was no effect on PCI-related myocardial injury. There was however, a significant increase in death, stroke and non-fatal MI at one year — 18.9% with IL-1 inhibition versus 5.4% in control (Morton et al., 2015), questioning this therapeutic approach following AMI.

Interestingly, it has been recently shown in a large phase III Canakinumab Anti-inflammatory Thrombosis Outcomes Study (CANTOS) clinical trial (NCT01327846), that quarterly subcutaneous injections of ACZ885 (also known as canakinumab, a monoclonal antibody against IL-1β) in combination with standard of care prevented recurrent cardiovascular events (CV death, non-fatal MI, and non-fatal stroke) over a median follow-up of 3.8 year among 10,061 people with prior myocardial infarction and with a high-sensitivity C-reactive protein (hsCRP) level of ≥ 2 mg/L. Whether the administration of canakinumab to AMI patients to target IL-1β to dampen the pro-inflammatory response to AMI can reduce MI size and prevent adverse LV remodeling is not known.

#### 3.5.2 IL-6

A recently published clinical study has demonstrated that an intravenous infusion of Tocilizumab (humanized anti-IL-6R antibody that binds to both membrane-bound and soluble IL-6R), reduced peri-procedural myocardial injury in NSTEMI patients undergoing PCI in terms of less serum high-sensitive Troponin-T and CRP (Kleveland et al., 2016) (Table 1). The ongoing ASSAIL-MI clinical study is currently investigating the effects of this therapeutic approach on MI size in STEMI patients treated by PPCI (ClinicalTrials.gov Identifier: NCT03004703).

### 3.6 Why have so many anti-inflammatory therapies failed to protect in AMI?

The reason why so many anti-inflammatory therapeutic strategies have failed to reduce MI size or improve clinical outcomes in AMI patients, despite showing beneficial effects in small and large animal models of AMI, is not clear. A number of potential factors have been suggested:

(1) Animal models: The animal AMI models used to test potential cardioprotective therapies in the experimental setting are far removed from the typical AMI patient (reviewed in Ferdinandy, Hausenloy, Heusch, Baxter, & Schulz, 2014; Lecour et al., 2014). For example, the use of a rodent model in the study of inflammation is cautioned as the rodent has a much greater and more resilient inflammatory response than other species (Seok et al., 2013; Warren et al., 2010). Furthermore, the experimental models tend to use young healthy animals of same gender and similar genetic background, and induce acute coronary artery occlusion in a health coronary artery. In contrast, the typical AMI patient is middle aged, has one or more co-morbidities (such as diabetes, hypertension, and obesity which may interfere with cardioprotective therapies), is on co-medications (such as statins, beta-blockers, anti-platelet P2Y12 inhibitors which may interfere with cardioprotective therapies), and acute coronary artery occlusion occurs due to plaque rupture in an inflamed and diseased coronary artery;
(2) Anti-inflammatory efficacy: The therapy may have failed to adequately suppress the pro-inflammatory response in AMI patients, despite showing beneficial effects in animal AMI models. For example, the C5B inhibitor, Pexelizumab, failed to inhibit assembly of the terminal complement complex in STEMI patients undergoing PPCI, which may in part explain the lack of effect on clinical outcomes in the APEX-AMI trial (Martel et al., 2012).
(3) Inflammation as a therapeutic target: It has been suggested that the pro-inflammatory response to AMI may not contribute to ischemic cardiomyocyte death. Experimental studies in mice deficient in P-selectin and intercellular adhesion molecule-1 (Briaud et al., 2001), MCP-1 (Dewald et al., 2005), and animals with defective IL-1 signaling (Bujak et al., 2008), did not sustain a reduction in MI size, when compared to wild-type, despite having a suppressed inflammatory response following AMI.
(4) Study design and protocol: Selecting the right dose for testing in clinical studies is challenging, especially, when in many cases, phase 2 clinical studies were not undertaken. It is difficult to extrapolate doses shown to be effective in small and large animal studies, into clinical studies. Furthermore, in some clinical studies, the therapeutic agent was given at a different time-point compared to the experimental studies (in relation to the phases of acute ischemia and reperfusion). The timing of the anti-inflammatory agent is crucially important, depending on the intended target (i.e. neutrophils or cytokines and so on), and in this regard, further work is required to determine the optimum timing of the therapeutic agent. Another issue is the heterogeneity of patients in clinical trials, which of course is not reproduced in the experimental animal studies, and this may impact on sample size for showing an effect with a therapeutic agent in the clinical setting. Finally, patient selection is critical with patients presenting with shorter ischemic times (<3–4 h) most likely to benefit from a cardioprotective therapy administered at the time of reperfusion (Hausenloy, Botker, et al., 2017; Kloner et al., 2006).

As such, in order to improve the translation of future therapeutic strategies for preventing inflammation in AMI into the clinical setting more clinically relevant animal AMI models should be used and one should ensure that the therapy can adequately suppress the immune response following AMI. In addition, a multicenter blinded randomized placebo-controlled approach should be adopted in the pre-clinical assessment of potential cardioprotective strategies, in order to improve the translation of cardioprotective for patient benefit (Hausenloy, Garcia-Dorado, et al., 2017). One such initiative, the Consortium for preclinicAl assESsment of cARdioprotective therapies (CAESAR) was established to improve the pre-clinical evaluation of cardioprotective therapies using a multi-center (3 sites with small and large animal models) blinded randomized controlled studies with standardized acute IRI protocols and centralized analysis of data (Jones et al., 2015; Lefer & Bolli, 2011). However, with the exception of ischemic preconditioning, the CAESAR network failed to demonstrate multi-center cardioprotection with either sodium nitrite (Lefer et al., 2014) or sildenafil citrate (Kukreja et al., 2014), despite these agents showing cardioprotection in single-center studies. The reasons for the discordancy between the single and multi-center studies, with respect to these 2 interventions, are not clear, but may either be due to a true lack of cardioprotection in the multi-center setting with these 2 agents, or it may reflect the difficulties and challenges of standardizing animal husbandry and acute IRI protocols across the 3 different sites. As such, for any such multi-center cardioprotection research network to work in the future, will require careful co-ordination, rigorous selection of the cardioprotective agent to be tested, meticulous standardization of animal selection and care, and the acute IRI protocols.

### 3.7 Future therapeutic approaches to target inflammation

#### 3.7.1 Multi-targeted therapies

The reasons for the many failures to target inflammation as a strategy for cardioprotection are unclear but may relate to the limitations of using a single-targeted approach directed to only the pro-inflammatory proponent of acute myocardial IRI. This approach does not address the other components of acute myocardial IRI (for example mitochondrial dysfunction, calcium overload, oxidative stress, microvascular obstruction, other components of the inflammatory response and so on). As such, a multi-targeted approach and combining anti-inflammatory agents with mitochondria and endothelial protective therapies may be a better approach to reducing MI size in reperfused STEMI patients (Hausenloy, Garcia-Dorado, et al., 2017).

#### 3.7.2 TLR2

Experimental studies have demonstrated that administration of anti-TLR2 antibodies (OPN-301 or OPN-305) reduced MI size when administered at the time of reperfusion, in both small and large animal AMI models (Arslan et al., 2010, Arslan et al., 2012). A recent phase I study, has demonstrated safety with OPN-305 administered to healthy volunteers (Reilly et al., 2013), and phase II trials are currently underway to test the efficacy of OPN-305 in patients with myelodysplastic syndrome (ClinicalTrials.gov Identifier: NCT02363491) and patients undergoing renal transplantation (ClinicalTrials.gov Identifier:NCT01794663). It would be interesting to investigate whether targeting TLR2 with OPN-305, administered at the time of reperfusion can reduce myocardial inflammation and limit MI size in patients presenting with AMI.

#### 3.7.3 NLRP3 inflammasomes

Experimental rodent studies have shown that genetic and pharmacological inhibition of the NLRP3 inflammasome may reduce MI size and prevent adverse LV remodeling (Coll et al., 2015; Kawaguchi et al., 2011; Marchetti et al., 2014; Mezzaroma et al., 2011; Pomerantz et al., 2001; Sandanger et al., 2013). Crucially, pharmacological inhibition of the NLRP3 inflammasome (using 16673-34-0) has been shown to reduce MI size in the murine heart, when administered at time of reperfusion, and even up to 60 min after the onset of reperfusion (Toldo et al., 2016), making it an attractive therapeutic target in STEMI patients treated by PPCI. A more specific small molecule inhibitor of the NLRP3 inflammasome, MCC950, has been described but has not been tested in the setting of acute myocardial IRI (Coll et al., 2015). The NLR3P3 inflammasome inhibitor could be administered in combination with therapies capable of targeting early reperfusion injury for additive benefits in terms of MI size reduction. The next step in the translational pathway would be to test whether pharmacological inhibition of the NLRP3 inflammasome is effective in a clinically-relevant large animal MI model (such as the porcine heart).

#### 3.7.4 eRNA

The release of extracellular RNA following AMI is a potent mediator of inflammation (Zernecke & Preissner, 2016), and experimental small animal studies have reported that administering RNAse1 to reduce extracellular RNA can reduce MI size (Cabrera-Fuentes et al., 2014; Stieger et al., 2017). Whether RNAse1 can reduce MI size in a clinically-relevant large animal MI model (such as the porcine heart) is not known and remains to be tested.

## 4 Conclusion

The complex inflammatory response to AMI is orchestrated by a number of different players, the roles of which change, according to the pro-inflammatory and anti-inflammatory reparative phases which follow AMI. Elucidation of the mechanisms underlying the inflammatory response to AMI, has identified a number of therapeutic targets for reducing acute MI size and preventing adverse LV remodeling following AMI, based on suppressing the pro-inflammatory phase, and upregulating the anti-inflammatory response. However, a number of anti-inflammatory therapies have failed to reduce MI size, and improve clinical outcomes in AMI patients. Improving the translation of anti-inflammatory therapies from the experimental to the clinical setting may be achieved by a number of approaches including: selecting the appropriate target at the right time or even combining multiple targets or broad-spectrum approaches appropriate for a multi-player process; identifying the broader timing issue of not only ischemia versus reperfusion but rather inhibiting the acute phase while enhancing the later reparative phase; appropriate power and design of clinical trials; more effective vetting of candidate therapies through rigorous preclinical trial assessment.

## Conflict of interest

All authors declare no actual or potential conflict of interest.
